# Resistance to commonly used insecticides and underlying mechanisms of resistance in *Aedes aegypti* (L.) from Sri Lanka

**DOI:** 10.1186/s13071-020-04284-y

**Published:** 2020-08-10

**Authors:** H. Sachini D. Fernando, Karla Saavedra-Rodriguez, Rushika Perera, William C. Black, B. G. D. Nissanka Kolitha De Silva

**Affiliations:** 1 Center for Biotechnology, Department of Zoology, Faculty of Applied Sciences, University of Sri Jayewardenepura, Nugegoda, Sri Lanka; 2 Department of Microbiology, Immunology and Pathology, Colorado State University, Fort Collins, CO 80523 USA

**Keywords:** *Aedes aegypti*, Insecticide resistance, Pyrethroid, Temephos, *kdr*, Metabolic resistance, Sri Lanka

## Abstract

**Background:**

Drastic increases of dengue fever (DF) over the past few years have prompted studies on the development of resistance to insecticides in the mosquito vector, *Aedes aegypti* (Linnaeus). In Sri Lanka control of the vector population is essentially achieved using larvicides (temephos) and adulticides (principally pyrethroids). The present study investigates resistance to commonly used insecticides and underlying mechanisms of *Ae. aegypti* in selected sites in Sri Lanka.

**Methods:**

In this study, susceptibility to three commonly used adulticides (malathion, permethrin and deltamethrin) and the larvicide temephos were tested for *Ae. aegypti* sampled from five localities in Sri Lanka using WHO dose diagnostics tests. In addition, we performed dose-response tests for permethrin to determine lethal concentrations (LCs) with CDC bottle bioassays. An assessment of the activity of metabolic detoxifying enzymes (multifunction oxidases (MFOs), glutathione S-transferases (GSTs) and esterases) and determination of frequency of the *kdr* mutations (F1534C, V1016G and S989P) were also carried out to ascertain the associated resistance mechanisms. *Kdr* genotype frequencies were compared with samples collected from the same sites in 2015 to determine the change of allele frequencies over the years.

**Results:**

The present study revealed resistance in all *Ae. aegypti* populations studied, with low mortality percentages for both permethrin (10–89%) and deltamethrin (40–92%). Dose response tests revealed highest resistance ratios (RR) for permethrin and temephos from Colombo district whereas Puttalum district exhibited the lowest. High frequencies of the 1534C allele (0.052–0.802) were found in the study sites in 2017. Comparison with samples collected in 2015 revealed a substantial increase in this allele. The activity of MFOs and *p-*nitro phenyl-acetate esterase was significantly greater in most Sri Lankan populations in comparison to that of the New Orleans (NO) susceptible strain. In contrast, the activity of α-esterase and β-esterase was similar or lower than that in the NO strain.

**Conclusions:**

*Aedes aegypti* from Sri Lanka is resistant to pyrethroid insecticides showing rapid selection for *kdr* mutations and varying metabolic mechanisms. Continued monitoring of vector populations is crucial to mitigate the development of resistance to commonly used insecticides and in turn, controlling the vector population.
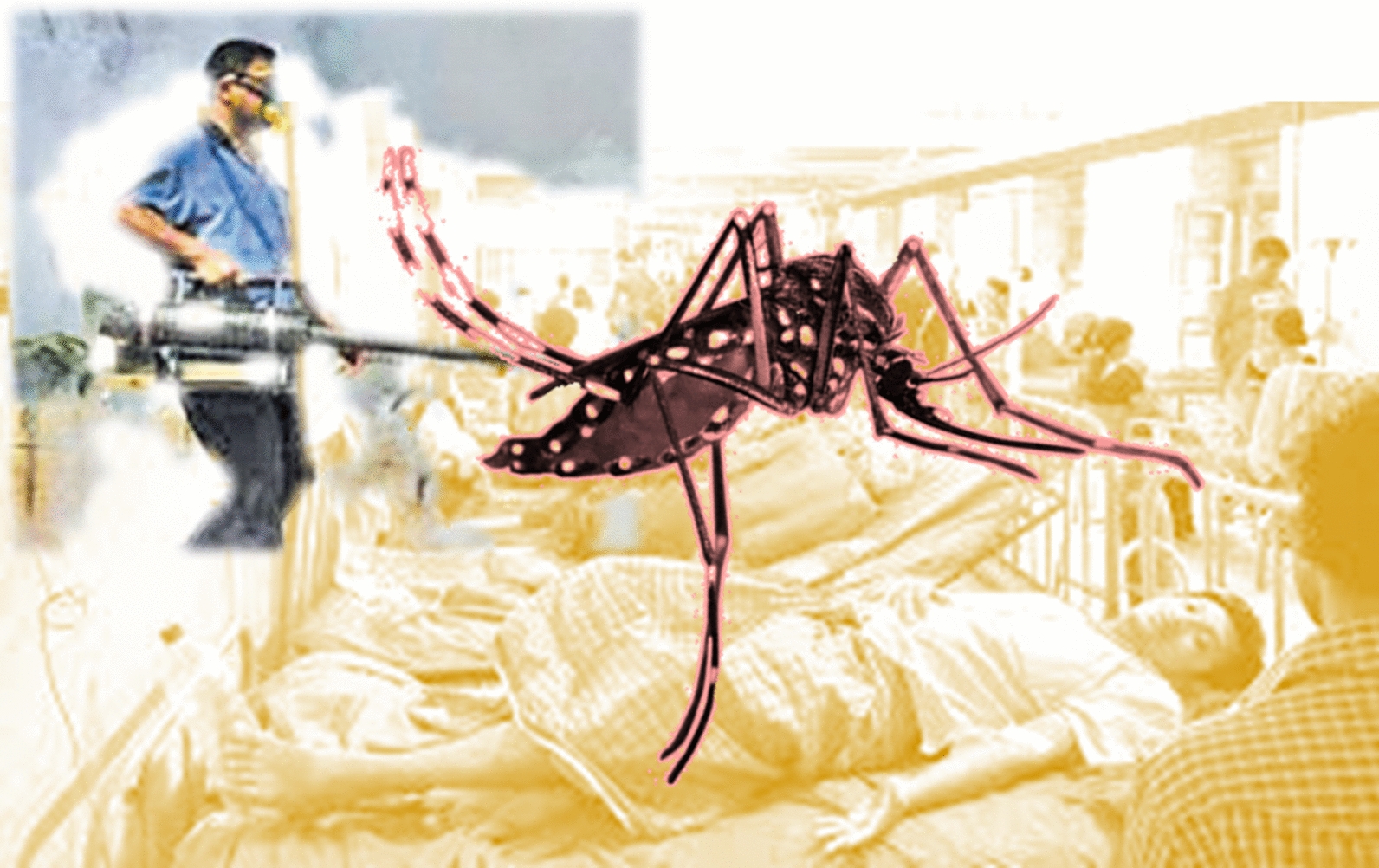

## Background

Mosquito-borne arboviruses have become a major primary health concern, due to the rapid global increase in prevalence [1]. Dengue is a severe viral infection and dengue virus (DENV) is transmitted by *Aedes* mosquitoes. Approximately 50% of the worlds’ population is at risk of dengue transmission, making it one of the most important arboviral diseases of the world [2, 3]. Although the dengue vaccine Dengvaxia, by Sanofi Pasteur (CYD-TDV) and the yellow fever vaccine (17DD and 17D-204 YF) have been registered for use against two of the most serious arboviral diseases, dengue and yellow fever, most of prevention and control of these arboviral diseases depend on the vector control measures undertaken in most of the endemic countries [4–6]. Typically, dengue control relies on vector control through elimination of the breeding places and the reduction of vector populations through the application of insecticides.

In Sri Lanka, dengue fever (DF) has become an important communicable disease, with an alarming increase in the number of reported cases. In 2017 alone, 185,688 DF cases were reported to health authorities, with 45% of the cases being reported from the Western Province, the main commercial province in Sri Lanka [7]. This number represents a 4.7-fold increase in the average number of cases observed annually in the years 2010 through 2016. Although the numbers decreased in the year 2018 with only 51,659 recorded DF cases, in the year 2019, 105,049 DF cases were reported to the health ministry of Sri Lanka [7]. Dengue transmission in Sri Lanka has been strongly correlated with rainfall [8]. Two distinct periods of DENV transmission occur annually, and these periods occur in association with the monsoon rains. The first and the largest DENV transmission period occurs from June to August and corresponds with the Southwest Monsoon. The second and the smaller period occurs from October to December and coincides with the Northeast Monsoon rainfall [8].

The reduction in DENV transmission in Sri Lanka is primarily achieved through vector control strategies. *Aedes aegypti* is the main target of these vector control strategies. The public is advised to eliminate potential breeding places around houses, and failure to do so can results in a monetary fine. Chemical adulticides in the form of fogging and larvicides are used extensively for vector management. Upon notification of a case or an outbreak, the health authorities apply Abate® (temephos) to the potential larval breeding sites and PestGuard^®^ 161 (d-tetramethrin 4% and cyphenothrin 12%) as an adulticide [9]. PestGuard^®^ 161 has been used as an adulticide since 2009, and it replaced the liquid form of malathion that was employed previously [9]. Use of mosquito coils, repellents, and vaporizers with pyrethroids as the active ingredient have become popular in households to control the vector.

Resistance development against commonly used insecticides has become a major dilemma in controlling many insect vectors of disease, compromising vector control programmes [10, 11]. The development of resistance to a particular insecticide has been associated with multiple resistance mechanisms. These mechanisms involve decreased cuticular penetration, behavioral modifications, increased detoxification, and decreased sensitivity of the target site. The two main mechanisms of resistance are increased detoxification using metabolic enzymes and the decreased sensitivity of the target sites [12]. Increased detoxification through metabolic enzymes is principally associated with three major enzyme groups, i.e. cytochrome P450 monooxygenases (P450s), esterases and glutathione S-transferases (GSTs). Single or multiple mutations appearing in the voltage-gated sodium channel (*vgsc*) are responsible for the target site insensitivity. The over production of esterase enzymes is responsible for organophosphate and carbamate resistance, monooxygenases are involved in pyrethroid and organophosphate metabolism, and GSTs with elevated activity are functionally involved in all four classes of insecticide resistance, mainly including organophosphate and DDT. Single mutations in the *vgsc* gene are associated with a decrease in the target site sensitivities for pyrethroid insecticide leading to knockdown resistance (*kdr*). Knockdown resistance is a form of target site insensitivity and is often considered as a major mechanism responsible for the reduced susceptibility to pyrethroid insecticides in insects. In *Ae. aegypti*, 11 *kdr* mutations responsible for pyrethroid resistance have been identified [13–19]. These mutations vary in the geographical spread, frequency in the mosquito population, and the effect it has on the resistance phenotype. However only five of these mutations have been linked to a functional resistance to pyrethroids. These include F1534C, V1016G, S989P, I1011M [20], and recently, V410L [19]. From these *kdr* mutations, F1534C is the most geographically widespread mutation, whereas mutations occurring at the 1016 position have a distinct geographical distribution. For example, V1016G mutation has been found in Asia whereas V1016I has been found in the Americas [21]. Recent studies conducted in three districts in Sri Lanka have suggested the presence of three *kdr* mutations, F1534C, V1016G and S989P. The study specifically targeted the pyrethroid-resistant *Ae. aegypti* mosquitoes and revealed the presence of three mutations [22, 23], thus implying the selection of *kdr* mutations in the mosquito populations. A study conducted in 2012 to understand the spraying efficacy of adulticides and larvicides revealed that deltacide® and Pestguard® could be successfully used as adulticides and temephos as a larvicide to control the mosquito populations in Sri Lanka [9]. Several studies have reported the susceptibility status of *Ae. aegypti* mosquitoes breeding in drain water and brackish water in Sri Lanka [24, 25]. A study analyzing the susceptible status of the drain-dwelling *Ae. aegypti* in Sri Lanka reported that mosquitoes with elevated activities of GST and monooxygenases were resistant to pyrethroids, malathion and propoxur [24]. *Aedes aegypti* collected from brackish water breeding sites were reported as resistant to propoxur (0.1%), whereas no significant differences were observed when these brackish water breeders were compared with fresh water breeders for permethrin (0.25%) and malathion (4%) [25].

Monitoring resistance to commonly used insecticides and understanding the mechanisms that contribute to the particular resistance are critical in the management of disease transmitting vectors and the spread of communicable diseases. However, the development of resistance against commonly used insecticides in mosquitoes has created a serious obstacle to the control of the *Ae. aegypti* populations below a threshold that is capable of transmitting DENV. Although several studies have been carried out in the country regarding the insecticide resistance in *Ae. aegypti* populations, a comprehensive study recognizing the associated mechanisms that contribute to the resistance is lacking. The present study evaluates the resistance status of commonly used insecticides and their associated mechanisms in five sampling sites in five districts in Sri Lanka. A study site per district was selected, based on the number of DF cases recorded and the frequency of insecticide application over the past years. Thus, the present study was designed to bridge the gap in knowledge of the resistance status of *Ae. aegypti* populations in Sri Lanka against commonly used insecticides in the country and to investigate the underlying resistance mechanisms involved.

## Methods

### Mosquito sampling and rearing

**Fig. 1:**
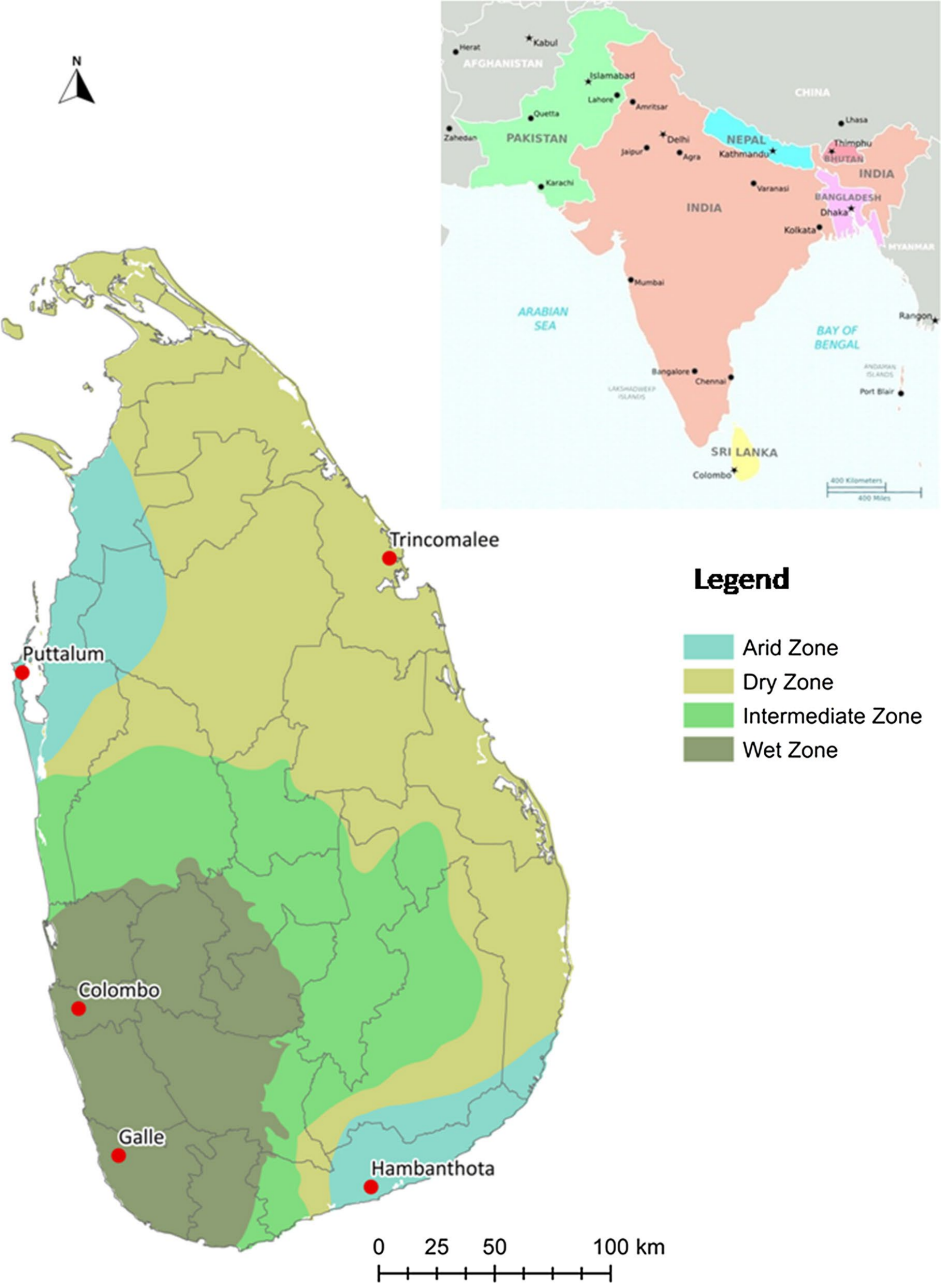
Map of Sri Lanka displaying the sampling locations. Red dots indicate the sampling sites and the grey lines indicate the district boundaries on the map

Wild mosquitoes in preimaginal stages were sampled with ovitraps in 2017 from five districts. The geographical locations (Fig. 1) and the description of each site are provided in Table 1. Approximately 100 ovitraps were laid in the five districts. The collection sites were selected with a focus on residential areas that recorded DF cases frequently. The traps were collected five days later. Larvae were also collected from containers that held water at the time of inspection. The immature stages (field and F_0_ generation) were collected and taken to the laboratory and fed on fish feed. After emergence, the adults were sorted morphologically to the species level using standard taxonomic keys [26]. *Aedes aegypti* females of the F_0_ generation were artificially blood-fed to induce egg-laying. The larval offspring of the resulting F_1_ were fed on fish food, whereas adults were fed on a 10% sucrose solution. The mosquito cages were housed in an insectary at room temperature at 27 ± 2 °C, with a relative humidity of 80–100% and a light: dark photoperiod of 12 h:12 h. From each of the geographical collections 2 subsamples of 48 unfed female mosquitoes were stored at − 80 °C for *kdr* genotyping and biochemical activity processing. The remaining mosquitoes of the F_1_ generation were used for bioassays. The experiment was repeated with the New Orleans (NO) strain which was used as an insecticide susceptible reference strain (Colorado State University, USA). A laboratory strain (SriLanka_Lab) that had been reared in the insectarium of the University of Sri Jayewardenepura, Sri Lanka, for 30 generations without any exposure to insecticides was also used in the experiments.

**Table 1:** Collection sites with geographical coordinates and a description of the study sites in Sri Lanka

Sampling district	Geographical coordinates of study sites		Description of study sites	Frequency of insecticide application
	Latitude	Longitude		
Colombo	6°51′16″	79°54′11″	Urban, populated area	Frequent
Galle	6°47′00″	79°58′00″	Urban, populated area	Frequent
Hambanthota	6°01′00″	80°46′60″	Suburban, semi-populated area	Not frequent
Puttalum	8°14′00″	79°46′00″	Suburban, populated area	Not frequent
Trincomalee	8°37′00″	81°13′00″	Urban, populated area	Frequent

### Adult bioassay

Susceptibility of adult *Ae. aegypti* mosquitoes to permethrin, deltamethrin and malathion were evaluated using the standard WHO mosquito bioassay protocol with WHO standard tubes and insecticide impregnated papers containing diagnostic dosages (Universiti Sains, Malaysia) [23]. Three to five-day-old, sucrose-fed females were used for the susceptibility tests. Experiments were conducted in 4 replicates and included a control with 25 mosquitoes per tube for each insecticide for each sampling location.

Test mosquitoes were exposed for 1 h to an impregnated filter paper containing the WHO prescribed diagnostic dose (deltamethrin 0.05%, permethrin 0.75% and malathion 5%). Control experiments consisted of a filter paper impregnated with the solvent of the particular insecticide. After the 1 h exposure mosquitoes were transferred to holding tubes, provided with 10% w/v sucrose solution and the final mortality was recorded at 24 h post-exposure. Collections were categorized according to the resulting mortality percentage with the use of WHO guidelines: 98–100% mortality denotes susceptibility; 90–97% mortality denotes possible resistance; and less than 90% mortality denotes resistance.

### Bottle bioassay for the determination of LC_50_

The susceptibility of the Sri Lankan *Ae. aegypti* mosquitoes towards the insecticide permethrin was calculated with the use of the bottle bioassay method [27]. Permethrin in 7 different doses was tested to provide a stock solution with a concentration of 0.5, 1.0, 2.5, 5.0, 10.0, 15.0 and 20.0 µg/ml. A solution of 1 ml from each diagnostic dose was added to a 250 ml Wheaton bottle, spread evenly inside the surface, and left to dry overnight. Newly emerged, 3–4-day-old, female mosquitoes not yet blood-fed were introduced in batches of 20 to bottles coated with each dose of permethrin. The experiment was replicated four times. Knockdown was recorded every 10 min up to 1 h and the mosquitoes were then transferred to holding cups and provided with a 10% w/v sucrose solution. Final mortality was recorded after 24 h of exposure. The results were analyzed with a binomial logistic regression model using the IRMA Qcal software (http://sourceforge.net/projects/irmaproj/) to determine the 50% and 90% lethal concentrations (LC_50_ and LC_90_). Resistance ratios (RR_50_ and RR_90_) were calculated by comparing LC_50_ and LC_90_ rates of the field sampled populations with the LC_50_ and LC_90_ rates of the susceptible New Orleans strain.

### Larval bioassays for temephos

Bioassays to determine larval susceptibility were performed according to the WHO recommendations. The late second and early third-instar larvae were used for the experiments. Susceptibility of the larvae to the temephos insecticide was tested using 5 concentrations (0.002–0.04 µg/ml). Four replicates were tested per concentration, and 5 concentrations were included in the experimental setup, with 25 larvae per replicate and concentration. The insecticide concentrations were adjusted to obtain different mortality percentages ranging from 0–100%. The mortality of larvae was recorded after 24 h of exposure. The results were analyzed with IRMA Qcal software to determine the 50% (LC_50_) and 90% (LC_90_) lethal concentrations for temephos. Resistance ratios (RR_50_ and RR_90_) were calculated by comparing the LC_50_ and LC_90_ rates of the field sampled populations with the LC_50_ and LC_90_ rates of the susceptible New Orleans strain.

### Genotyping for the detection of *kdr* mutation

A total of 336 females, which were not exposed to the insecticide bioassays were examined in the 2017 sampling. These samples consisted of 48 females from each collection site (Table 1, Fig. 1) and were subject to DNA extraction [28]. DNA samples were analyzed to detect the F1534C, V1016G and S989P genotype frequencies in each population. The most commonly occurring *kdr* mutations (F1534C, V1016G and S989P) in the *vgsc* gene were genotyped in 48 F1 mosquitoes using allele specific PCR (AS-PCR) and melting curve conditions [29, 30].

Similarly, F1534C, V1016G and S989P mutations in the *vgsc* gene were analyzed in random *Ae. aegypti* samples collected in 2015 from the same localities as given in Table 1 and Fig. 1. These samples have been previously collected through ovitraps and BG-1 Sentinel traps and a maximum of 5 individuals per trap have been selected to avoid sampling of homogeneous populations [31].

Genotype frequencies at F1534C, locus was calculated against the Hardy-Weinberg equilibrium (HWE) using the Chi-square goodness-of-fit test with 1 degree of freedom, as it was the only reporting mutation. The formula: F_IS_ = 1 – (H_obs_/H_exp_), where H_obs_ and H_exp_ denote the number of observed heterozygotes and the expected number of heterozygote genotypes, respectively, was used to calculate Wright’s inbreeding coefficient (F_IS_). An excess of homozygotes of the mutant allele is expressed when F_IS_ ≥ 0, an excess of mutant heterozygotes is indicated with F_IS _< 0, and the 95% confidence interval was calculated as ± Z_0.05_ √*p*(1−*p*)/*n* [32].

### Biochemical assays

Biochemical assays to detect altered enzymatic activity were conducted according to the method described by Valle et al. [33]. Forty-seven *Ae. aegypti* females, representing each sampling site and not exposed to insecticide assays were homogenized individually in 300 µl of cold deionized water. The activities of multifunction oxidases (MFOs) glutathione S-transferases (GSTs), α-esterases, β-esterases, *p*-nitrophenyl acetate esterases (*p*NPA), acetylcholinesterase (AChE), inhibited acetylcholinesterase (iAChE), and total proteins were quantified colorimetrically.

The heme peroxidation method was used to measure the activity of MFO. TMBZ (Sigma-Aldrich, St. Louis, MO, USA) was used as a substrate. The reaction was conducted in microtiter plates in duplicate, in a total volume of 305 µl. The reaction contained 20 µl of the supernatant, 17.7 mM potassium phosphate buffer (pH 7.2), 0.03% TMBZ/123 mM sodium acetate buffer (pH 5.0), and 0.25% H_2_O_2_. Cytochrome C in 250 mM sodium acetate, pH 5.0 (0.01 mg/ml)) was used as the positive control, while potassium phosphate buffer (90 mM, pH 7.2) was used as the negative control. Absorbance was read at 650 nm after 90 min of incubation. General oxidase (and heme) content was expressed as micrograms of cytochrome per milligram protein using varying quantities of cytochrome C obtained from bovine heart (Sigma-Aldrich).

A reaction mixture containing 195 µl of a reduced form of glutathione (GSH)/CDNB solution (9.5 mM GSH in 100 mM potassium phosphate buffer pH 6.5/1 mM CDNB in methanol) was added to the microwell plate to measure the level of GST. This mixture was incubated for 1 h and the absorbance was measured at 340 nm at 1 min intervals for 20 min.

α- and β-esterases hydrolyze α- or β-naphthyl acetate to produce naphthol which forms diazo-dy complexes in the presence of Fast Blue B. The reaction mixture contained 200 µl of either α- or β-naphthyl acetate/sodium phosphate mix (0.3 mM α- or β- naphthyl acetate in 20 mM sodium phosphate buffer pH 7.2) and 10 µl of centrifuged homogenates. This reaction was incubated for 15 min at room temperature followed by the addition of 50 µl of Fast Blue (0.3% Fast Blue in 3.5% SDS; Sigma-Aldrich). The mixture was then incubated at room temperature for 5 min. α- or β-naphthol 3.5 nmol/µl was used as the positive control. Absorbance was read at 405 nm at 15 s intervals for 2 min.

For the quantification of the acetylcholinesterases, 25 µl of homogenate was added to two microplates, marked as ACHE and iACHE with a final volume of 205 µl per well, which included, 145 µl of Triton/Na phosphate (1% Triton X-100 in 100 mM sodium phosphate buffer (pH 7.8)) and 10 µl of 10 mM DTNB in 100 mM sodium phosphate buffer (pH 7.0). Acetylcholine iodine with 0.3 mM propoxur was introduced to the plate labeled AChE and acetylcholine iodine without propoxur was introduced to the plate labeled iAChE. Each plate containing the mixture was read at 405 nm after the 1 h incubation period. The iAChE results are expressed as a percentage of the remaining AChE activity after the addition of propoxur.

The Bradford assay was used to quantify the proteins, with a 1:5 dilution of Bio-Rad (Bio-Rad, Hercules, CA, USA) protein assay dye concentrate in water, which was examined in comparison to 1 µg/ml bovine serum albumin (BSA) (Sigma Aldrich). The plate containing the reaction mix was read at 620 nm after an incubation period of 5 min.

### Statistical analysis

Normality of the data was confirmed using a Shapiro-Wilk test with the *P*-value set at 0.05. Means and standard errors were calculated using the *emmeans* package in R. A one-way ANOVA and Tukey pair-wise comparison was used to compare activity means between populations using the *car* and *emmeans* package in R, respectively.

A series of Pearson correlation analyses was conducted for the following: (i) insecticide (permethrin) LC_50_ values and *kdr* allele frequency; (ii) insecticides (permethrin and temephos) LC_50_ and mean enzymatic activity; (iii) insecticide % mortality (permethrin and deltamethrin) and *kdr* allele frequency; and (iv) insecticide % mortality (permethrin, deltamethrin and malathion) and mean enzymatic activity. The data were fitted to a linear model, and a Pearson correlation test was performed using the *cor.test* in R. Additionally, Pearson correlation coefficients were estimated within the pyrethroids, within the organophosphates and between the two classes of insecticides.

## Results

The susceptibility of the mosquito collections from the five sampling sites to WHO discriminating dosages of permethrin (0.75%), deltamethrin (0.05%) and malathion (5%) was assessed based on the mortality percentage of mosquitoes after the 24 h exposure (Table 2). The mosquitoes collected from all localities showed some degree of resistance to permethrin and deltamethrin with mortalities varying between 10–89% and 40–92%, respectively. The highest mortality percentage for the pyrethroids (permethrin and deltamethrin) was recorded from the Puttalum district, (permethrin, 89%; deltamethrin, 92%) whereas the lowest mortality percentage was reported from the Colombo district (permethrin, 10%; deltamethrin, 40%). All Sri Lankan *Ae. aegypti* populations were susceptible to malathion. The SriLanka_Lab strain exhibited 100% mortality for the three insecticides tested.

**Table 2:** Mean mortality percentages for the pyrethroid and organophosphate insecticides in the *Aedes aegypti* populations

Population	Mean mortality % (*n* = 100)		
	Permethrin	Deltamethrin	Malathion
Colombo	10	40	97
Galle	67	79	93
Puttalum	89	92	100
Trincomalee	72	64	98
Hambanthota	75	82	99
SriLanka_Lab	100	100	100

The LC_50_ and LC_90_ values and the resistance ratios (RR) for permethrin and temephos are shown in Table 3. The RR values were calculated using the New Orleans mosquito strain. The highest LC_50_ or LC_90_ value for permethrin was recorded for the Colombo district with a RR_50_ of 12.9 and RR_90_ of 9.62 in comparison with the NO strain. The lowest LC_50_ value for permethrin was recorded in the Puttalum district (1.80 (1.57–2.05)). This population also recorded the lowest RR_50_ and RR_90_ compared with NO (RR_50_ = 2.6, RR_90_ = 3.11). The results also indicated that all Sri Lankan *Ae. aegypti* populations exhibited high degrees of resistance when using the NO strain for comparison. The resistance ratios (RR_50_) for temephos varied between 0.69 for the Puttalum district and 3.93 in the districts of Galle and Trincomalee. The mosquitoes collected from the Puttalum district were more susceptible than the NO strain to temephos.

**Table 3:** Diagnostic doses for temephos and permethrin determined using the New Orleans strain

Population	LC_50_ (95% CI)	Resistance ratio (RR_50_)	LC_90_ (95% CI)	Resistance ratio (RR_90_)
Temephos				
Colombo	0.0167 (0.0145–0.0192)	3.88	0.0414 (0.0318–0.0538)	6.47
Galle	0.0169 (0.0155–0.0184)	3.93	0.036 (0.031–0.042)	5.63
Puttalum	0.0030 (0.0026–0.0033)	0.69	0.0071 (0.006–0.008)	1.11
Trincomalee	0.0169 (0.0143–0.0199)	3.93	0.0434 (0.029–0.063)	6.78
Hambanthota	0.0112 (0.0103–0.0121)	2.60	0.0210 (0.0179–0.0245)	3.28
New Orleans	0.0043 (0.0040–0.0045)	1.0	0.0064 (0.0059–0.0070)	1.00
Permethrin				
Colombo	8.75 (7.84–9.98)	12.9	15.88 (12.52–17.98)	9.62
Galle	5.04 (4.23–5.99)	7.4	17.30 (13.10–22.86)	10.48
Puttalum	1.80 (1.57–2.05)	2.6	5.13 (3.92–6.72)	3.11
Trincomalee	4.42 (3.87–5.05)	6.5	11.42 (8.88–14.69)	6.92
Hambanthota	5.84 (5.24–6.52)	8.6	10.52 (8.75–13.49)	6.38
New Orleans	0.6783 (0.6078–0.7569)	1.0	1.65 (1.26–2.16)	1.00

*Abbreviations*: LC, lethal concentration; CI, confidence interval

**Fig. 2:**
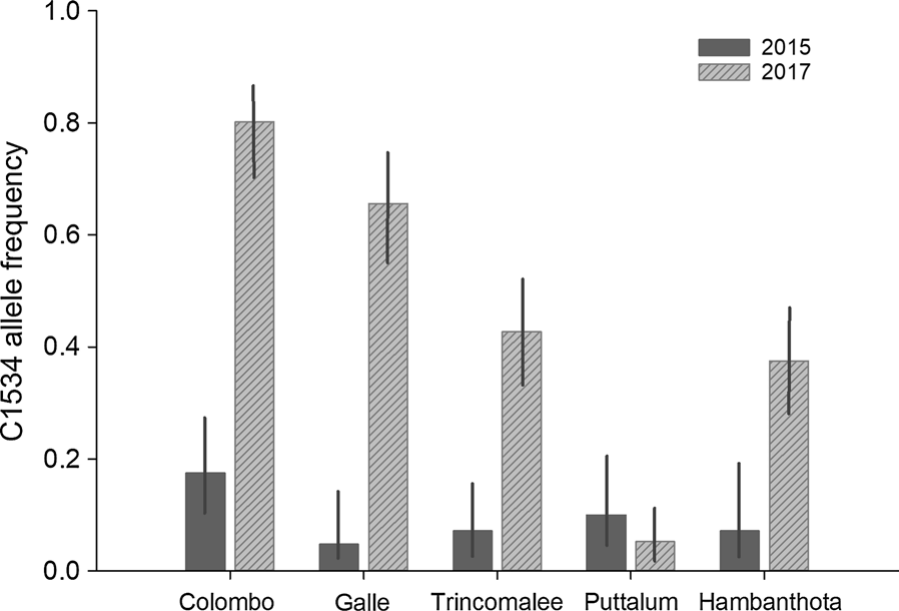
Increase of the 1534C mutant allele frequency in *Ae. aegypti* collections in 2015 and 2017

To evaluate the mode of resistance of *Ae. aegypti* to permethrin and deltamethrin, the frequencies of the three reported *kdr* mutant alleles in the *vgsc* gene were estimated. The 1534C mutant allele was recorded in all the populations studied, and the V1016G and S989P mutations were not recorded in any of the populations. The mutant allele frequencies for 1534C ranged between 0.048–0.175 in the 2015 collections, whereas two years later in the 2017 collections, the frequency of 1534C increased dramatically from 0.052 to 0.802 (Table 4, Fig. 2). Interestingly the mosquitoes collected from the Colombo district in 2017 showed a rapid increase in the 1534C mutant allele compared to the frequency of that allele in the 2015 collections (0.175–0.802). However, the Puttalum district collection showed a decrease of the 1534C allele between the two collections (0.100 to 0.052). The highest 1534C allele frequency for both 2015 and 2017 collections was recorded from the Colombo district whereas the lowest was recorded from the Puttalum district (0.052) in the 2017 collections and, in the district of Hambanthota (0.071) and Trincomalee district (0.071) in the 2015 collections. It was of interest to note that the SriLanka_Lab strain exhibited a 1534C allele frequency of 0.229. Thus, the SriLanka_Lab strain was not used for the RR comparisons.

**Table 4:** Genotypes detected for F1534C mutation in *Aedes aegypti* populations for the 2015 and 2017 samples respectively

Population	Total no. of mosquitoes	F1534C genotype			Resistant C allele frequency	Chi-square	F_IS_
		F/F	F/C	C/C			
2015 collection							
Colombo	39	30	6	3	0.175	0.002	0.480
Galle	21	19	2	0	0.048	0.818	− 0.05
Trincomalee	28	25	2	1	0.071	0.014	0.461
Puttalum	30	26	2	2	0.100	0.001	0.629
Hambanthota	21	18	3	0	0.071	0.000	− 0.076
2017 collection							
Colombo	48	1	17	30	0.802	0.423	− 0.115
Galle	47	7	17	23	0.656	0.205	0.164
Trincomalee	48	19	15	14	0.427	0.004	0.352
Puttalum	47	42	5	0	0.052	0.622	− 0.103
Hambanthota	47	23	12	12	0.375	0.000	0.448
New Orleans	48	48	0	0	0.000	0.000	0.000
Sri Lanka_Lab	48	30	14	4	0.229	0.000	0.174

**Fig. 3:**
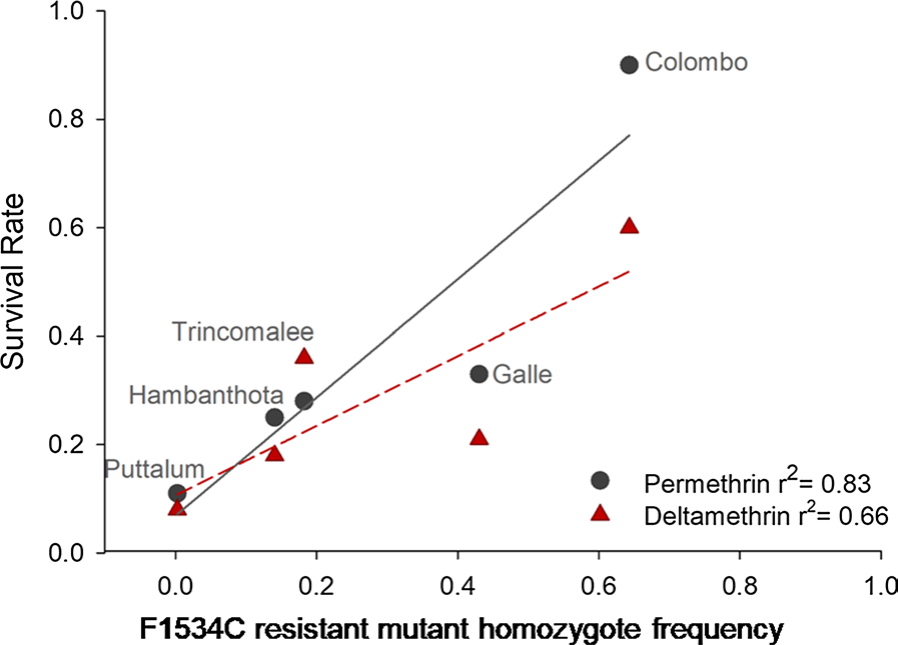
Plot of survival as a function of the expected frequency (HWE) of 1534C mutant allele homozygotes from all five collection sites. The vertical lines correspond to 95% confidence intervals

Figure 3 is a plot of survival as a function of the expected frequency of the 1534C homozygotes (HWE). Even though the correlations for both permethrin (*r*^2^ = 0.83) and deltamethrin (*r*^2^ = 0.66) were large and positive, few points exist to assess significance.

**Fig. 4:**
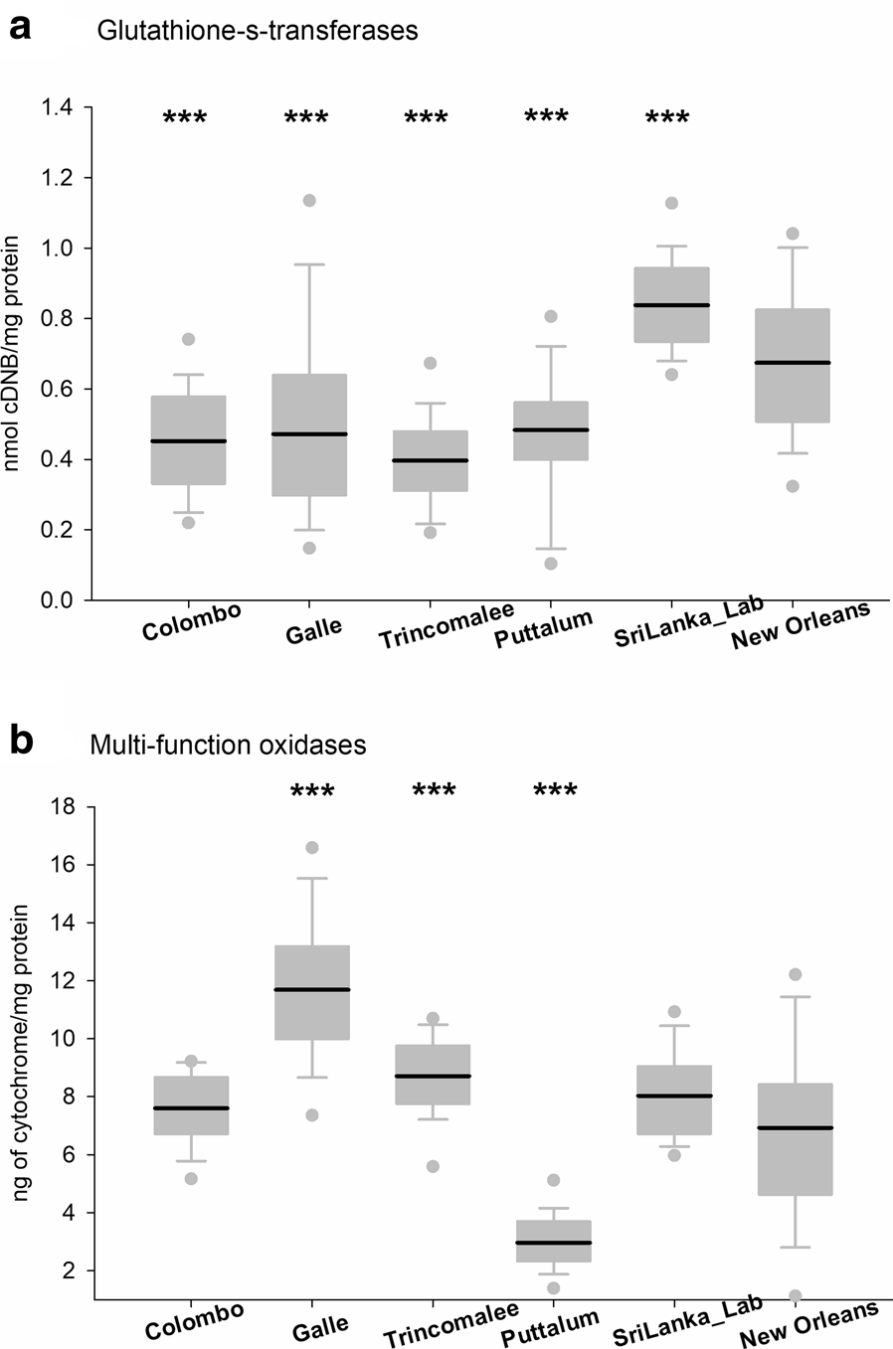
Enzyme activity for *Aedes aegypti* collected from Sri Lanka. **a** Glutathione-S-transferases. **b** Multifunction oxidases. Boxes include the mean (dark line), 10th, 25th, 75th and 90th percentiles. Asterisks indicate a significant difference between the New Orleans and the corresponding strain by a t-test pairwise comparison with pooled SD (*P < 0.05, **P < 0.005, ***P < 0.0005)

**Fig. 5:**
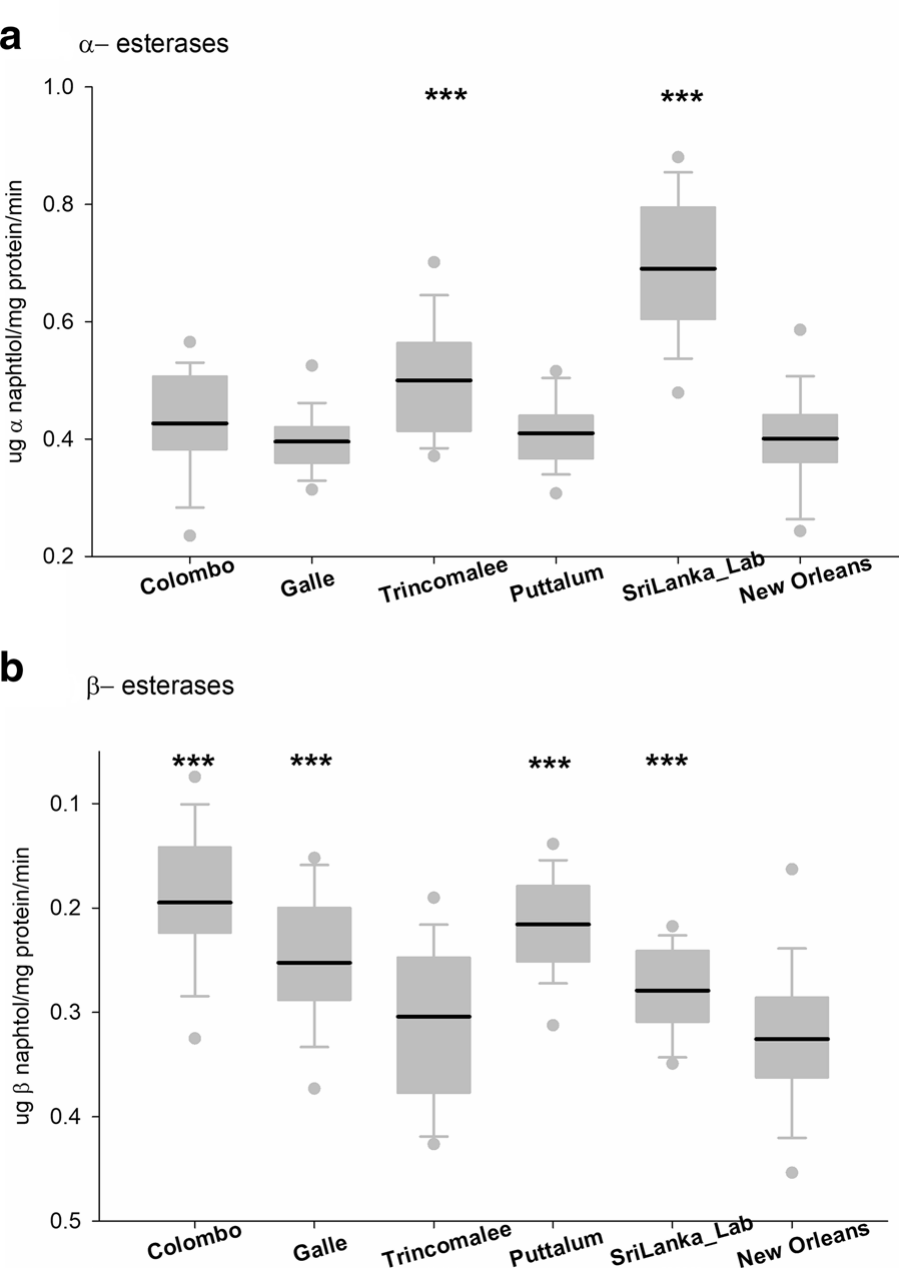
Enzyme activity for *Aedes aegypti* collected from Sri Lanka. **a** Alpha-esterases. **b** Beta- esterases. Boxes include the mean (dark line), 10th, 25th, 75th and 90th percentiles. Asterisks indicate a significant difference between the New Orleans and the corresponding strain by a t-test pairwise comparison with pooled SD (*P < 0.05, **P < 0.005, ***P < 0.0005)

**Fig. 6:**
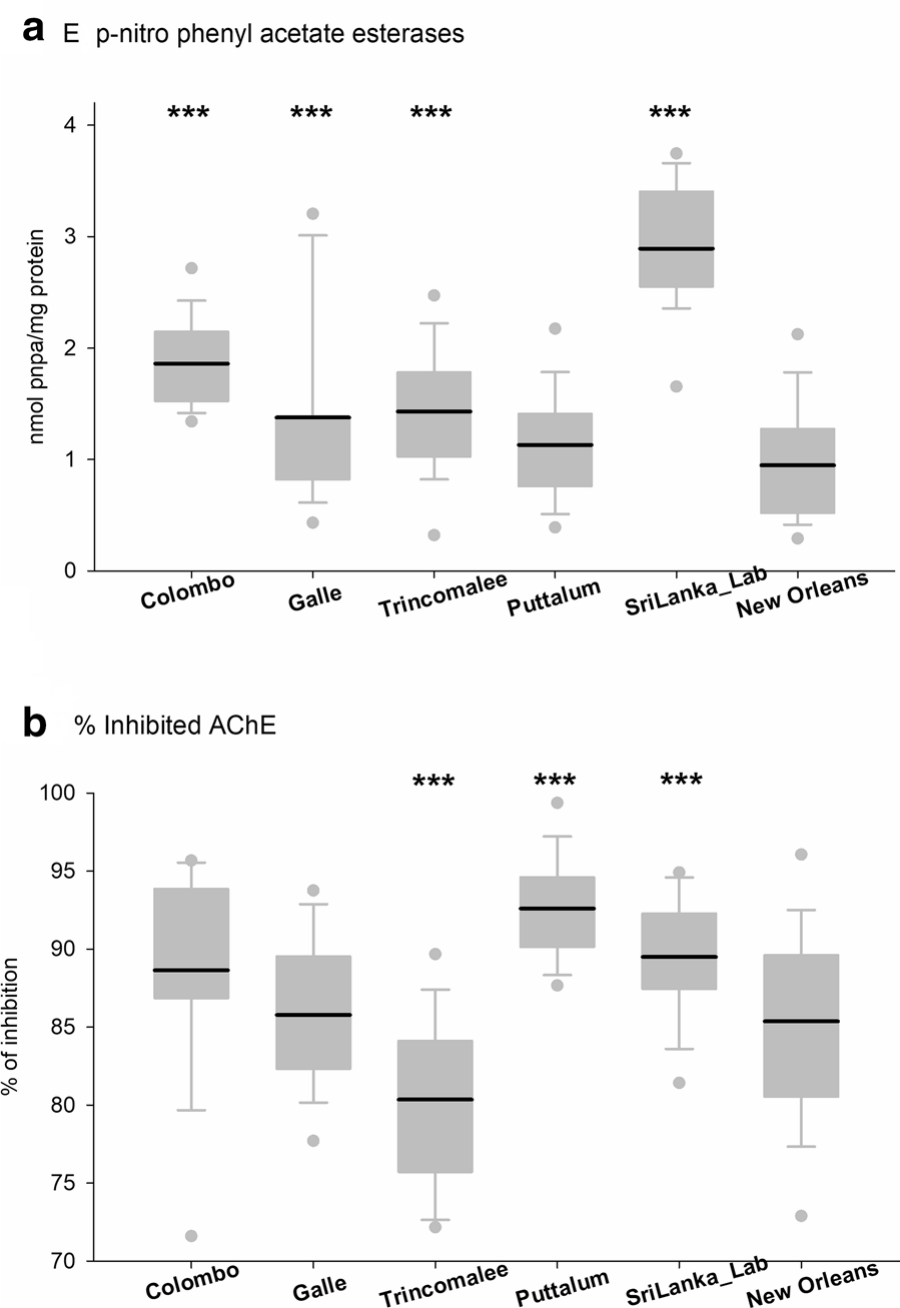
Enzyme activity for *Aedes aegypti* collected from Sri Lanka. **a** p-nitrophenyl acetate esterases. **b** Inhibited acetyl cholinesterase obtained. Boxes include the mean (dark line), 10th, 25th, 75th and 90th percentiles. Asterisks indicate a significant difference between the New Orleans and the corresponding strain by a t-test pairwise comparison with pooled SD (*P < 0.05, **P < 0.005, ***P < 0.0005)

Altered enzymatic activity was assessed through general biochemical assays (Figs. 4, 5 and 6). Although the highest GST activity was observed in the SriLanka_Lab strain followed by the NO strain, significant difference was not observed between any of the four collections from Sri Lanka and the laboratory strain. The highest MFOs were recorded from the Galle district whereas the lowest were recorded from Puttalum district. SriLanka_Lab strain and NO showed similar MFOs. The highest α-esterases activity was observed in the SriLanka_Lab strain followed by the collection from Trincomalee. The collections from Galle and Puttalum showed similar α-esterases activity to that of the NO strain. The β-esterases were highest in the Colombo district collection followed by the Puttalum collections. The lowest β-esterases were recorded from the NO strain. The activity of *p*NPA was highest in the SriLanka_Lab strain, whereas the lowest was recorded in the NO strain. The remaining activity of iAcHE was higher in the Sri Lankan collections than in the NO strain, except for the Trincomalee collection.

The correlation analysis between the enzymatic activity and the mortality percentages for the three adulticides (Table 5) tested revealed positive correlations between the *p*NPA enzymatic activity with permethrin and deltamethrin. All three adulticides had negative correlation with the MFO activity tested.

**Table 5:** Correlation analyses between mortality percentages and the biochemical activity in *Aedes aegypti* populations for the 2017 samples

	MFO	α-esterases	β-esterases	GST	*p*NPA	iAChE
Permethrin	− 0.27 (*P* = 0.662)	− 0.14 (*P* = 0.823)	0.65 (*P* = 0.238)	0.54 (*P* = 0.349)	− 0.97 (*P* = 0.007)*	− 0.08 (*P* = 0.893)
Deltamethrin	− 0.30 (*P* = 0.621)	− 0.45 (*P* = 0.443)	0.47 (*P* = 0.422)	0.70 (*P* = 0.192)	− 0.97 (*P* = 0.007)*	0.16 (*P* = 0.800)
Malathion	− 0.86 (*P* = 0.065)	0.14 (*P* = 0.825)	0.23 (*P* = 0.716)	0.43 (*P* = 0.467)	− 0.54 (*P* = 0.343)	0.22 (*P* = 0.722)

*Significant correlations

Cross-resistance analysis (Table 6) reveals positive correlation between permethrin and deltamethrin (*r*^2^ = 0.9453, *P*-value = 0.0044) although no correlation was observed between the pyrethroids and temephos or the pyrethroids and malathion.

**Table 6:** Cross-resistance analysis for *Aedes aegypti* populations for the 2017 samples in Sri Lanka

	Permethrin	Deltamethrin	Malathion	Temephos
Deltamethrin	0.9453 (*P* = 0.0044)*			
Malathion	0.4284 (*P* = 0.3967)	0.3772 (*P* = 0.4610)		
Temephos	− 0.6870 (*P* = 0.1315)	− 0.7836 (*P* = 0.0652)	− 0.7482 (*P* = 0.0872)	

*Significant correlations

*Notes*: Mortality rates were used for the permethrin–deltamethrin and malathion. Resistance ratios were used for the temephos

## Discussion

Mosquitoes sampled from five localities in Sri Lanka were analyzed to determine the level of their susceptibility to pyrethroids (permethrin and deltamethrin) and organophosphates (malathion and temephos (larvicide)). Additionally, the LC_50_ for permethrin was estimated by the bottle bioassay method to compare the levels of resistance in adult *Ae. aegypti* populations. The present study revealed that Sri Lankan mosquitoes were resistant to pyrethroid adulticides, to both permethrin and deltamethrin and organophosphorus larvicide, temephos according to the WHO criteria. The LC_50_ values for permethrin confirmed these results. Additionally, the bottle bioassay and WHO methodologies were highly correlated for the populations tested with permethrin (*r*^2^ = 0.919, *P* = 0.0096). As the same populations were subjected to both CDC bottle bioassay experiment and WHO methodologies to test the resistance for permethrin, this revealed the similarity between the two methods. Interestingly, Colombo showed a mortality of 10% in the WHO test and a RR of 12.9 using the bottle bioassay test.

By testing different collection sites, we identified a significant correlation between permethrin and deltamethrin mortality rates (*r*^2 ^= 0.9453, *P* = 0.004) but not between the RR and mortality rates of the organophosphates temephos and malathion (*r*^2 ^= −0.7482, *P* = 0.0872), respectively. As expected, no correlation was observed between the pyrethroids and temephos or the pyrethroids and malathion, indicating cross-resistance is not present between classes of insecticides.

Analysis of *kdr* mutations in the *vgsc* gene revealed the presence of 1534C mutant allele in mosquitoes collected in the years 2015 and 2017. A substantial increase of the 1534C allele was observed in 2017. Interestingly, no mutant alleles were observed in the 1016 or 989 positions of the *vgsc* gene.

According to the published literature, the increased levels of resistance to pyrethroids can be correlated to the presence of *kdr* mutant alleles in the natural populations [13, 34]. Electrophysiological tests conducted on wild samples, in addition to the samples with induced mutagenesis, have confirmed that the *kdr* mutations found in *Ae. aegypti* alter the sensitivity to pyrethroid insecticides [35, 36]. The present study detected an alarming increase of the 1534C *kdr* mutant alleles in the study sites between 2015 and 2017. Although the degree of susceptibility to pyrethroid insecticides in the mosquitoes collected in 2015 from locations is unknown, the present study revealed an elevated degree of resistance in all the *Ae. aegypti* samples collected in 2017.

Previous studies in three localities in Sri Lanka have reported the presence of the 1016G and 989P mutant allele in the pyrethroid-resistant *Ae. aegypti* populations at exceptionally low frequencies [23]. Interestingly, the present study failed to identify either of these mutations in the populations studied. Possibly, the V1016G and S989P mutations are rare in the wild mosquito populations as the previous publication specifically genotyped pyrethroid-resistant *Ae. aegypti* mosquitoes [23]. In the present study, randomly selected mosquito populations were analyzed to detect the *kdr* mutations to avoid bias toward the resistant phenotype. The 1534C mutation is far more common than the 1016G mutation in the wild populations [37]. Recent studies in Mexico have revealed the co-existence of *kdr* mutations with a higher level of phenotypic resistance [38, 39]. A sequential model has been proposed, wherein the 1534C mutation occurs first, thereby initiating the occurrence of resistance and the mutation at the 1016 position is thought to follow and provide higher levels of resistance [38]. It has also been suggested that 1534C allele may have a lower fitness cost relative to the mosquitoes with 989P + 1016G mutant alleles [40, 41]. The results of the present study corroborate with the hypothesis as the 1534C mutation has not disappeared in the Sri Lankan laboratory colony (SriLanka_Lab), albeit it has not been exposed to insecticides for over 30 generations. The low frequency of homozygotes of the 1534 C/C detected in the laboratory colony also explained the susceptibility of SriLanka_Lab to the WHO test as *kdr* resistance is exhibited by a recessive genotype. Recent studies carried out in the Asian region showed high frequencies of 1534C mutant allele when compared with the frequency of 989P + 1016G mutant alleles reported [41]. It has been also suggested that F1534C mutation have multiple origins and in Asian populations V1016G mutant haplotype and S989P + V1016G haplotype have emerged from a F1534C haplotype [42]. Our analysis of *Ae. aegypti* from the 2015 collections revealed the presence of the F1534C mutation, thus indicating a population that may have been resistant to type I pyrethroids [23]. Studies carried out regarding the distribution of *kdr* alleles in Southeast Asia have revealed the presence of the S989P, V1016G/I, I1011M/V and F1534C mutations in the mosquito populations [43]. In Southeast Asian countries including Indonesia, Myanmar, Thailand and Malaysia, the V1016G mutation has been detected co-occurring with the S989P mutation. In Myanmar the co-occurrence of triple mutations V1016G, S989P and F1534C has been detected. Although V1016I has been detected more commonly in North and South America, recent studies in Vietnam have revealed the presence of this mutation [43].

An excess of *kdr* homozygotes, 1534C mutant allele, in the Hardy-Weinberg analysis is responsible for the deviation from the natural equation. The observation of the high frequency of *kdr* homozygotes early in the evolution of resistance is very intriguing. The most likely explanation for this scenario would be that all the sites except Puttalum and Hambanthota have been continuously treated with pyrethroid insecticides over the years. This situation would have selected for *kdr* 1534C homozygotes since pyrethroid resistance is only expressed in homozygotes for the resistant alleles.

The metabolic activity of the Sri Lankan *Ae. aegypti* populations was compared with the NO susceptible strain. The Sri Lankan *Ae. aegypti* populations did not exhibit any differences in GST when compared with the NO strain. GST is an enzyme that is linked with resistance to DDT [44]. DDT was phased out for use in Sri Lankan mosquito control programs in the late 1970s and was replaced by malathion, and later by pyrethroid insecticides between 1995 and 1997 [45]. Thus, the lapse in the use of DDT for a long period may have resulted in susceptibility to DDT, thus explaining detection of the low frequency of GST in the collections studied. A high level of MFO activity was detected in Galle and Trincomalee. Oxidative enzymes are responsible for the metabolic resistance to permethrin [46]. The results of the present study reveal that the elevated degree of pyrethroid resistance perceived in the mosquito populations could be a combined effect from both the high level of *kdr* mutations observed and the elevated level of MFOs. Interestingly the Puttalum district which had the lowest resistance to pyrethroid insecticides, also showed the lowest MFO activity. The insecticide usage in the Puttalum district is exceptionally low; thus, the present results confirm the susceptibility of the mosquito populations to the currently used insecticides. For all the esterases analyzed, the Sri Lankan populations had high *p*NPA activity compared to the NO strain. However, α- and β-esterase activity revealed no significant difference in any of the Sri Lankan populations compared to the NO strain. Increased esterases in insects are known to be related to organophosphate resistance [12]. Although resistance to the organophosphate adulticide malathion was not recorded in the current study, resistance to temephos, which is an organophosphate larvicide, was recorded from all the study sites. Increased activity of esterases has been linked with chlorpyriphos and temephos resistance in *Ae. aegypti* sampled from Venezuela [46], Trinidad [47] and Cuba [48]. Although temephos resistance has been linked to increased levels of esterases, the specific esterase and inhibitors have not been identified [49]. Increased MFO activity has been suggested to play a partial role in pyrethroid resistance as well with *kdr* acting as the major mechanism [50].

We performed a Pearson correlation test between the mean activity of the enzymes and the mortality rates (WHO) obtained in different populations of Sri Lanka. Significant correlation was identified between permethrin resistance and *p*NPA activity (*r*^2 ^= −0.97, *P* = 0.007) and between deltamethrin resistance and *p*NPA activity (*r*^2 ^= −0.97, *P* = 0.007). The role of *p*NPA enzymes in pyrethroid resistance is not completely understood but metabolism of pyrethroid by esterases has been demonstrated in many insects [51].

Resistance to commonly used insecticides has been investigated in brackish water breeding as well as in drain-water breeding *Ae aegypti* mosquitoes in Sri Lanka [24, 25]. Drain-water breeding mosquitoes were reported to be resistant to pyrethroids (deltamethrin and permethrin) and to show higher GST and MO activities [24]. In the brackish water study although the resistant levels were not statistically significant between brackish water- and freshwater-derived mosquitoes for propoxur and permethrin, brackish water mosquitoes were more resistant to malathion [25].

Studies carried out in Cuba showed that *Ae. aegypti* populations resistant to temephos also exhibited increased levels of resistance to deltamethrin [52, 53]. This scenario has also been observed in *Ae. aegypti* populations exhibiting resistance to a pyrethroid (permethrin) after developing resistance to temephos [48].

According to WHO criteria, the study sites exhibited an increase in temephos resistance in *Ae. aegypti*. Typically, temephos (Abate) is applied extensively in dengue epidemic areas to reduce the number of *Aedes* larvae, especially in large water-containing receptacles or as an application in the potential breeding places. However, the widespread and unplanned application of temephos has led to the development of resistance in most of the study sites. The discriminating dose for temephos recommended by the WHO is 0.012 mg/l [54], and all study sites reported resistance to this dosage. Resistance to temephos has been recorded in many countries in Asia, including Laos, Cambodia, Thailand and Vietnam [55–58]. A study carried out in Rio de Janeiro and Espirito Santo, Brazil, uncovered resistance to temephos in *Ae. aegypti* populations with the mortality percentages ranging from 23.5% to 74% [59]. Apart from the inefficiency due to the built-up resistance, temephos is known to be toxic to aquatic and non-target organisms [60].

In Sri Lanka, the control of dengue currently relies on community participation for the lessening of mosquito breeding places and the application of insecticides for adult and larval mosquito populations. Although the community participation is an important part of vector control programs, the failure to remove water-collecting containers and proper disposal of waste has led to an increase in mosquito breeding sites. Although massive attempts have been made to reduce dengue through community participation, the extensive use of chemical insecticides and a persistent increase in the incidence of dengue illustrate the futility of the employed management methods. However, it should be noted that the present study was carried out in one sampling site per each district, in which the sampling sites and the districts were selected according to the reported DF cases. Resistance status of the mosquito populations may differ in different sites in the same district depending on the ecological and geographical factors, frequency of application of insecticides, mosquito movement etc. Thus, a more elaborative study covering all ecological and geographical distributions in the country would be more beneficial. The monitoring of the resistance in vectors in Sri Lanka and new vector control strategies, including insecticides effective for the targeted vectors, urgently need to be implemented. The novel alternatives should cost less and should not harm non-target organisms.

## Conclusions

The present study indicated resistance to pyrethroid insecticides and temephos in Sri Lankan *Ae. aegypti* populations. This finding was confirmed by both modification to the *vgsc* gene and altered enzymatic activity. The results obtained were in congruence with the susceptibility bioassay results and biochemical results obtained for the previous studies carried out in Sri Lanka. The results also indicate that the use of malathion as an adulticide could be proven efficient to control the vector populations. Importantly, the study suggests the need for continuous monitoring of resistance to commonly used insecticides and of their underlying mechanisms.

## Data Availability

All data generated and/or analyzed during this study are included in this published article.

## Abbreviations

DF: Dengue fever
*Kdr*: Knockdown resistance
*Vgsc*: Voltage gated sodium channel
AS-PCR: Allele specific polymerase chain reaction
MFO: Multifunction oxidases
GST: Glutathione S-transferases
*p*NPA: p-Nitrophenyl acetate esterases
AChE: Acetylcholinesterase
iAChE: Inhibited acetylcholinesterase
RR: Resistance ratio
LC: Lethal concentration

## References

[1] WHO (2009). Dengue: guidelines for diagnosis, treatment, prevention, and control.

[2] Murray NE, Quam MB, Wilder-Smith A (2013). Epidemiology of dengue: past, present and future prospects. Clin Epidemiol..

[3] Mayer SV, Tesh RB, Vasilakis N (2017). The emergence of arthropod-borne viral diseases: a global prospective on dengue, chikungunya and zika fevers. Acta Trop..

[4] Achee NL, Gould F, Perkins TA, Reiner RC, Morrison AC, Ritchie SA, Gubler DJ, Teyssou R, Scott TW (2015). A critical assessment of vector control for dengue prevention. PLoS Negl Trop Dis..

[5] Guy B, Barrere B, Malinowski C, Saville M, Teyssou R, Lang J (2011). From research to phase III: preclinical, industrial and clinical development of the Sanofi Pasteur tetravalent dengue vaccine. Vaccine..

[6] Staples JE, Gershman M, Fischer M, Centers for Disease Control and Prevention (CDC) (2011). Yellow fever vaccine: recommendations of the Advisory Committee on Immunization Practices (ACIP). MMWR Recomm Rep..

[7] Epidemiology Unit, dengue update. http://www.epid.gov.lk. Accessed 24 May 2019.

[8] Surendran SN, Veluppillai T, Eswaramohan T, Sivabalakrishnan K, Noordeen F, Ramasamy R (2018). Salinity tolerant *Aedes aegypti* and *Ae. albopictus* - infection with dengue virus and contribution to dengue transmission in a coastal peninsula. J Vector Borne Dis..

[9] Karunaratne SHPP, Weeraratne TC, Perera MDB, Surendran SN (2013). Insecticide resistance and, efficacy of space spraying and larviciding in the control of dengue vectors *Aedes aegypti* and *Aedes albopictus* in Sri Lanka. Pestic Biochem Physiol.

[10] Sarwar M, Salman M (2015). Insecticides resistance in insect pests or vectors and development of novel strategies to combat its evolution. Int J Bioinf Biomed Eng..

[11] Coleman M, Hemingway J, Gleave KA, Wiebe A, Gething PW, Moyes CL (2017). Developing global maps of insecticide resistance risk to improve vector control. Malar J..

[12] Hemingway J, Ranson H (2000). Insecticide resistance in insect vectors of human disease. Annu Rev Entomol..

[13] Brengues C, Hawkes NJ, Chandre F, McCarroll L, Duchon S, Guillet P (2003). Pyrethroid and DDT cross-resistance in *Aedes aegypti* is correlated with novel mutations in the voltage-gated sodium channel gene. Med Vet Entomol..

[14] Saavedra-Rodriguez K, Urdaneta-Marquez L, Rajatileka S, Moulton M, Flores AE, Fernandez-Salas I (2007). A mutation in the voltage-gated sodium channel gene associated with pyrethroid resistance in Latin American *Aedes aegypti*. Insect Mol Biol..

[15] Chang C, Shen WK, Wang TT, Lin YH, Hsu EL, Dai SM (2009). A novel amino acid substitution in a voltage-gated sodium channel is associated with knockdown resistance to permethrin in *Aedes aegypti*. Insect Biochem Mol Biol..

[16] Srisawat R, Komalamisra N, Eshita Y, Zheng M, Ono K, Itoh TQ (2010). Point mutations in domain II of the voltage-gated sodium channel gene in deltamethrin-resistant *Aedes aegypti* (Diptera: Culicidae). Appl Entomol Zool..

[17] Yanola J, Somboon P, Walton C, Nachaiwieng W, Prapanthadara LA (2010). A novel F1552/C1552 point mutation in the *Aedes aegypti* voltage-gated sodium channel gene associated with permethrin resistance. Pestic Biochem Phys..

[18] Kushwah RBS, Dykes CL, Kapoor N, Adak T, Singh OP (2015). Pyrethroid-resistance and presence of two knockdown resistance (*kdr*) mutations, F1534C and a novel mutation T1520I, in Indian *Aedes aegypti*. PLoS Negl Trop Dis..

[19] Haddi K, Tomé HV, Du Y, Valbon WR, Nomura Y, Martins GF (2017). Detection of a new pyrethroid resistance mutation (V410L) in the sodium channel of *Aedes aegypti*: a potential challenge for mosquito control. Sci Rep..

[20] Du Y, Nomura Y, Zhorov BS, Dong K (2016). Sodium channel mutations and pyrethroid resistance in *Aedes aegypti*. Insect..

[21] Moyes CL, Vontas J, Martins AJ, Ng LC, Koou SY, Dusfour I (2017). Contemporary status of insecticide resistance in the major *Aedes* vectors of arboviruses infecting humans. PLoS Negl Trop Dis..

[22] Hegoda WK, Fernando HS, De Silva BG (2017). Detecting of knock down resistance (*kdr*) F1534C allele in the dengue vector *Aedes aegypti* in peri-urban areas of Colombo South, Sri Lanka. J Entomol Zool Stud..

[23] Fernando SD, Hapugoda M, Perera R, Saavedra-Rodriguez K, Black WC, De Silva NK (2018). First report of V1016G and S989P knockdown resistant (*kdr*) mutations in pyrethroid-resistant Sri Lankan *Aedes aegypti* mosquitoes. Parasit Vectors..

[24] Surendran SN, Jayadas TT, Sivabalakrishnan K, Santhirasegaram S, Karvannan K, Weerarathne TC (2019). Development of the major arboviral vector *Aedes aegypti* in urban drain-water and associated pyrethroid insecticide resistance is a potential global health challenge. Parasit Vectors..

[25] Ramasamy R, Jude PJ, Veluppillai T, Eswaramohan T, Surendran SN (2014). Biological differences between brackish and fresh water-derived *Aedes aegypti* from two locations in the Jaffna peninsula of Sri Lanka and the implications for arboviral disease transmission. PLoS ONE..

[26] Belkin JN (1962). The mosquitoes of the South Pacific (Diptera, Culicidae).

[27] Brogdon W, Chan A (2010). Guideline for evaluating insecticide resistance in vectors using the CDC bottle bioassay.

[28] Ballinger-Crabtree ME, Black WC, Miller BR (1992). Use of genetic polymorphisms detected by the random-amplified polymorphic DNA polymerase chain reaction (RAPD-PCR) for differentiation and identification of *Aedes aegypti* subspecies and populations. Am J Trop Med Hyg..

[29] Yanola J, Somboon P, Walton C, Nachaiwieng W, Somwang P, Prapanthadara LA (2011). High-throughput assays for detection of the F1534C mutation in the voltage-gated sodium channel gene in permethrin-resistant *Aedes aegypti* and the distribution of this mutation throughout Thailand. Trop Med Int Health..

[30] Stenhouse SA, Plernsub S, Yanola J, Lumjuan N, Dantrakool A, Choochote W (2013). Detection of the V1016G mutation in the voltage-gated sodium channel gene of *Aedes aegypti* (Diptera: Culicidae) by allele-specific PCR assay, and its distribution and effect on deltamethrin resistance in Thailand. Parasit Vectors..

[31] Fernando HS, Hapugoda M, Perera R, Black WC, De Silva BG (2020). Gene flow patterns among *Aedes aegypti* (Diptera: Culicidae) populations in Sri Lanka. Insects..

[32] Agresti A, Coull BA (1998). Approximate is better than “exact” for interval estimation of binomial proportions. Am Stat..

[33] Valle D, Valle D, Montella IR, Medeiros PFV, Ribeiro RA, Martins AJ, Martins AJ (2006). Quantification methodology for enzyme activity related to insecticide resistance in *Aedes aegypti*.

[34] Du Y, Nomura Y, Satar G, Hu Z, Nauen R, He SY, Zhorov BS, Dong K (2013). Molecular evidence for dual pyrethroid-receptor sites on a mosquito sodium channel. Pro Natl Acad Sci..

[35] Scott JG (2019). Life and death at the voltage-sensitive sodium channel: evolution in response to insecticide use. Annu Rev Entomol..

[36] Chen M, Du Y, Wu S, Nomura Y, Zhu G, Zhorov BS, Dong K (2019). Molecular evidence of sequential evolution of DDT-and pyrethroid-resistant sodium channel in *Aedes aegypti*. PLoS Negl Trop Dis..

[37] Alvarez LC, Ponce G, Saavedra-Rodriguez K, Lopez B, Flores AE (2015). Frequency of V1016I and F1534C mutations in the voltage-gated sodium channel gene in Aedes aegypti in Venezuela. Pest Manag Sci..

[38] Vera-Maloof FZ, Saavedra-Rodriguez K, Elizondo-Quiroga AE, Lozano-Fuentes S, Black WC (2015). Coevolution of the Ile1016 and Cys1534 mutations in the voltage gated sodium channel gene of *Aedes aegypti* in Mexico. PLoS Negl Trop Dis..

[39] Saavedra-Rodriguez K, Maloof FV, Campbell CL, Garcia-Rejon J, Lenhart A, Penilla P (2018). Parallel evolution of *vgsc* mutations at domains IS6, IIS6 and IIIS6 in pyrethroid resistant *Aedes aegypti* from Mexico. Sci Rep..

[40] Fan Y, O’Grady P, Yoshimizu M, Ponlawat A, Kaufman PE, Scott JG (2020). Evidence for both sequential mutations and recombination in the evolution of *kdr* alleles in *Aedes aegypti*. PLoS Negl Trop Dis..

[41] Plernsub S, Stenhouse SA, Tippawangkosol P, Lumjuan N, Yanola J, Choochote W, Somboon P (2013). Relative developmental and reproductive fitness associated with F1534C homozygous knockdown resistant gene in *Aedes aegypti* from Thailand. Trop Biomed..

[42] Cosme LV, Gloria-Soria A, Caccone A, Powell JR, Martins AJ (2020). Evolution of *kdr* haplotypes in worldwide populations of *Aedes aegypti*: independent origins of the F1534C *kdr* mutation. PLoS Negl Trop Dis..

[43] Amelia-Yap ZH, Chen CD, Sofian-Azirun M, Low VL (2018). Pyrethroid resistance in the dengue vector *Aedes aegypti* in Southeast Asia: present situation and prospects for management. Parasit Vectors..

[44] Saavedra-Rodriguez K, Strode C, Flores AE, Garcia-Luna S, Reyes-Solis G, Ranson H (2014). Differential transcription profiles in *Aedes aegypti* detoxification genes following temephos selection. Insect Mol Biol..

[45] Karunaratne SH, Hemingway J (2001). Malathion resistance and prevalence of the malathion carboxylesterase mechanism in populations of mosquito vectors of disease in Sri Lanka. Bulletin WHO..

[46] Mazzarri MB, Georghiou GP (1995). Characterization of resistance to organophosphate, carbamate, and pyrethroid insecticides in field populations of *Aedes aegypti* from Venezuela. J Am Mosq Control Assoc..

[47] Vaughan A, Chadee DD, ffrench-Constant R. Biochemical monitoring of organophosphorous and carbamate insecticide resistance in *Aedes aegypti* mosquitoes from Trinidad. Med Vet Entomol. 1998;12:318–21 10.1046/j.1365-2915.1998.00111.x 9737606

[48] Bisset JA, Rodríguez MM, Ricardo Y, Ranson H, Perez O, Moya M, Vazquez A (2011). Temephos resistance and esterase activity in the mosquito *Aedes aegypti* in Havana, Cuba increased dramatically between 2006 and 2008. Med Vet Entomol..

[49] Wirth MC, Georghiou GP (1999). Selection and characterization of temephos resistance in a population of *Aedes aegypti* from Tortola, British Virgin Islands. J Am Mosq Control Assoc..

[50] Choovattanapakorn N, Yanola J, Lumjuan N, Saingamsook J, Somboon P (2017). Characterization of metabolic detoxifying enzymes in an insecticide resistant strain of *Aedes aegypti* harboring homozygous S989P and V1016G *kdr* mutations. Med Entomol and Zool..

[51] Bhatt P, Bhatt K, Huang Y, Lin Z, Chen S (2020). Esterase is a powerful tool for the biodegradation of pyrethroid insecticides. Chemosphere..

[52] Rodríguez MM, Bisset J, Ruiz M, Soca A (2002). Cross-resistance to pyrethroid and organophosphorus insecticides induced by selection with temephos in *Aedes aegypti* (Diptera: Culicidae) from Cuba. J Med Entomol..

[53] Rodríguez MM, Bisset JA, De Armas Y, Ramos F (2005). Pyrethroid insecticide-resistant strain of *Aedes aegypti* from Cuba induced by deltamethrin selection. J Am Mosq Control Assoc..

[54] WHO (1992). Vector Resistance to Pesticides, Fifteenth report of the WHO expert committee on Vector Biology and Control.

[55] Jirakanjanakit N, Rongnoparut P, Saengtharatip S, Chareonviriyaphap T, Duchon S, Bellec C (2007). Insecticide susceptible/resistance status in *Aedes* (Stegomyia) *aegypti* and *Aedes* (Stegomyia) *albopictus* (Diptera: Culicidae) in Thailand during 2003–2005. J Econ Entomol..

[56] Khun S, Manderson LH (2007). Abate distribution and dengue control in rural Cambodia. Acta Trop..

[57] Ranson H, Burhani J, Lumjuan N, Black IV WC. Insecticide resistance in dengue vectors. TropIK. 2010;1.

[58] Chareonviriyaphap T, Bangs MJ, Suwonkerd W, Kongmee M, Corbel V, Ngoen-Klan R (2013). Review of insecticide resistance and behavioral avoidance of vectors of human diseases in Thailand. Parasit Vectors..

[59] Lima EP, Paiva MHS, de Araújo AP, da Silva ÉVG, da Silva UM, de Oliveira LN (2011). Insecticide resistance in *Aedes aegypti* populations from Ceará. Brazil. Parasit Vectors..

[60] Vermeire T, Rikken M, Attias L, Boccardi P, Boeije G, Brooke D (2005). European union system for the evaluation of substances: the second version. Chemosphere..

